# Intraoperative ventilatory leak: Real‐time guidance for management of air leak in lung cancer patients undergoing VATS lobectomy

**DOI:** 10.1111/1759-7714.14925

**Published:** 2023-05-05

**Authors:** Gaetana Messina, Giovanni Natale, Mary Bove, Giorgia Opromolla, Vincenzo Di Filippo, Mario Martone, Antonio Noro, Beatrice Leonardi, Rosa Mirra, Francesca Capasso, Davide Gerardo Pica, Mario Grande, Francesco Panini D'alba, Giuseppe Vicario, Giovanni Liguori, Roberta Fiorito, Massimo Ciaravola, Eva Massimilla, Giovanni Messina, Alfonso Fiorelli, Giovanni Vicidomini, Fortunato Ciardiello, Morena Fasano

**Affiliations:** ^1^ Thoracic Surgery Unit Università degli Studi della Campania “Luigi Vanvitelli” Naples Campania Italy; ^2^ Anesthesiology and Intensive Care Unit Università degli Studi della Campania “Luigi Vanvitelli” Naples Campania Italy; ^3^ Otorhinolaryngology Unit Università degli Studi della Campania “Luigi Vanvitelli” Naples Campania Italy; ^4^ Nutrition Science University of Foggia Foggia Italy; ^5^ Oncology, Department of Precision Medicine Università della Campania “Luigi Vanvitelli” Naples Campania Italy

**Keywords:** lung resection, non‐small cell lung cancer, persistent air leaks (PAL), sealant, VATS lobectomy

## Abstract

**Background:**

Persistent air leak (PAL) is a common complication after video‐assisted thoracoscopic surgery (VATS) lobectomy. We aimed to evaluate whether the intraoperative quantitative measurement of air leaks using a mechanical ventilation test could predict PAL and identify those patients needing additional treatment for the prevention of PAL.

**Methods:**

This was an observational, retrospective, single‐center study that included 82 patients who underwent VATS lobectomy with a mechanical ventilation test for VL. Only 2% of patients who underwent lobectomy surgery had persistent air leaks.

**Results:**

At the end of lobectomy performed in patients with non‐small cell lung cancer, the lung was reinflated at a 25–30 mmH2O pressure and ventilatory leaks (VL) were calculated and in relation to the entity of the air leaks, we evaluated the most suitable intraoperative treatment to prevent persistent air leaks.

**Conclusion:**

VL is an independent predictor of PAL after VATS lobectomy; it provides a real‐time intraoperative guidance to identify those patients who can benefit from additional intraoperative preventive interventions to reduce PAL.

## Graphical abstract

Evaluation of intraoperative air leaks through the calculation of ventilatory leaks (VL) by mechanical ventilator during VATS lobectomy and identification of patients who may benefit from additional surgical treatments to prevent prolonged air leaks (PAL). VL is an independent predictor of PAL after VATS lobectomy; it provides an intraoperative guidance to identify those patients who can benefit from additional intraoperative preventive interventions to reduce PAL.

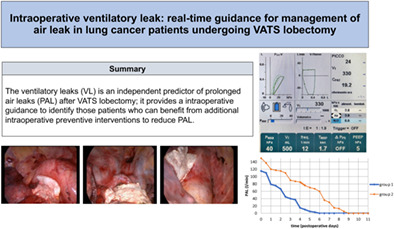

## INTRODUCTION

Intraoperative alveolar air leak (IOAAL) is one of the most frequent complications following video‐assisted thoracoscopic pulmonary lobectomy (VATS) surgery.

It occurs frequently and is due to injury to the pulmonary parenchyma, despite the development of “fissure‐last” techniques with the use of stapling devices to divide fissures.^1^, ^2^

The passage of air from the bronchial tree into the pleural space results in air leaks after pulmonary surgery. Bronchopleural fistula (BPF), originating from a more proximal airway or segmental bronchus, and alveolopleural fistula resulting from a leak distal to the segmental bronchus, are responsible for air leaks. Therefore, the most frequent causes of air leaks in pulmonary surgery are pleural alveolar fistulas, while BPF often require surgical intervention and have various risk factors. After pulmonary resection, air leakage is frequently right after surgery in about 60% of cases; however, most air leaks resolve within 48 h, although some will persist;^3^, ^4^ when the air leak lasts more than 5 days it is defined as a prolonged air leak; however, a fair heterogeneity of definitions is reported in the literature, and some authors use 7 days (or longer) as cutoff. Persistent air leaks (PAL) are reported with an incidence of 4%–16%.^5^ The identification of the air leak intraoperatively in real‐time thanks to the use of the mechanical ventilation test could assist us in the search for strategies to prevent PAL. The objective of our study was the evaluation of the association between intraoperative ventilatory leak (VL) and PAL after lung resection.

## METHODS

The study was led in compliance with the principles of the Declaration of Helsinki; written informed consent was obtained from all participants during preoperative communication and the protocol was approved by the Ethics Committee of the University of ‘Luigi Vanvitelli’ of Naples (ethical approval number: 833.18). This was an observational retrospective single‐center study whose primary aim was to measure intraoperative air leak in order to decide their intraoperative treatment.

We evaluated 82 patients—50 (61%) male, 32 (39%) female who were aged 66.4 ± 7.7 (years)—who underwent pulmonary lobectomy for non‐small cell lung cancer (NSCLC) from October 2019 to October 2022 at the Thoracic Surgery Department of the Vanvitelli University of Naples.

### Anesthesia and paravertebral block

All patients underwent echo‐guided paravertebral block ropivacaine 0.5% volume 20 mL.

General anesthesia was induced and maintained by total intravenous anesthesia with infusions of propofol and remifentanil and monopulmonary ventilation. Double lumen tube placement was guided using a flexible fiberscope, mechanical ventilation was initiated with a tidal volume of 5–6 mL/kg of predicted bodyweight, inspired oxygen of 0.4–0.5 and positive end‐expiratory pressure (PEEP) of 4–5 cmH2O were applied.

### Water immersion test

Lung resection surgery was performed by thoracoscopy with an anterior triportal approach according to Hansen.^6^ Lobectomy surgery was performed on monopulmonary ventilation, while intraoperative air leak assessment was performed on bipulmonary ventilation. After lobectomy, a warm sterile physiological saline solution was instilled into the thoracic cavity to check if the sutures of the bronchial stump and resection lines were airtight after each parenchymal resection through a water immersion test. We immersed all areas corresponding to the resection and dissection lines and carefully evaluated underwater, observing the degree of air bubbles, which formed during manual positive pressure ventilation, and searching for major air leaks. The anesthesiologist then gently reinflated the atelectatic lung slowly up to a peak pressure of 30 mmHg which was maintained for a few seconds.

### Intraoperative measurement of air leak

**FIGURE 1:**
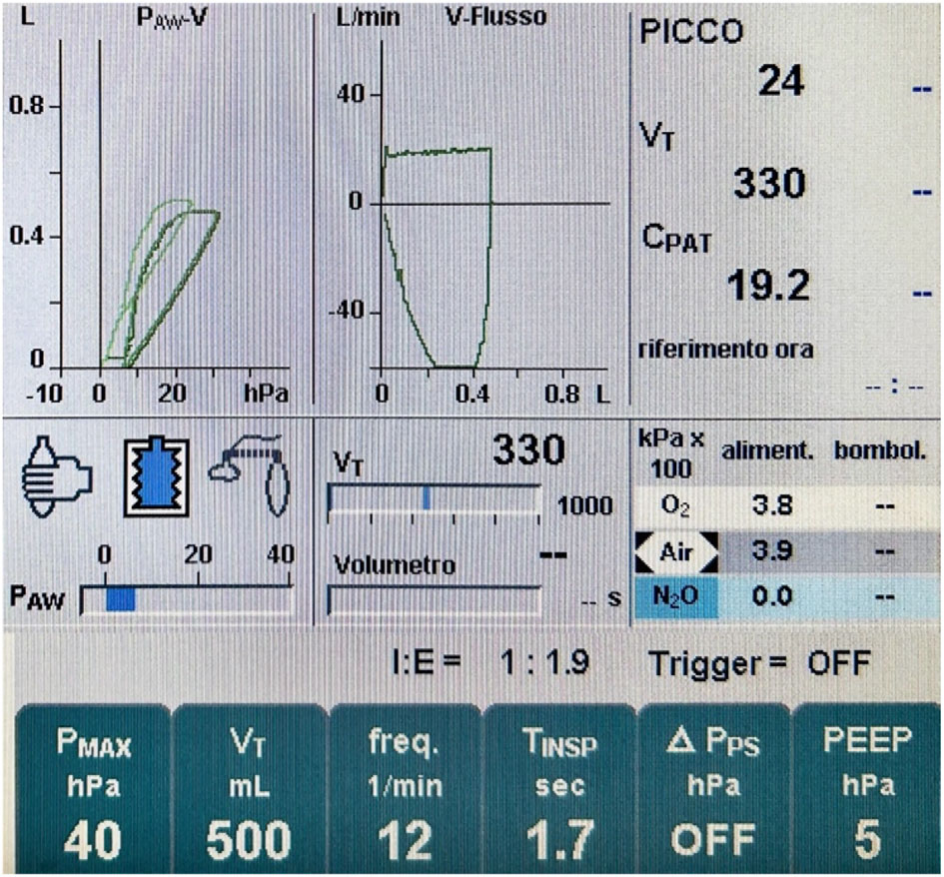
Ventilation mechanical test (VMT) after video‐assisted thoracoscopic surgery (VATS) lobectomy: inspiratory tidal volume − expiratory tidal volume/inspiratory tidal volume × 100.

The anesthetist performed the first intraoperative measurement of air leaks during the submersion test before the treatment. The anesthesiologist re‐expanded the lung with pressure of 25–30 cmH2O. The intraoperative alveolar air leaks (IOAAL) were calculated automatically by mechanical ventilation test, which came out as the difference between the inspiratory tidal volumes and expiratory measured during mechanical ventilation, using the following formula (inspiratory tidal volume − expiratory tidal volume/inspiratory tidal volume × 100) (Figure 1).

We classified IOAAL with the following cutoff:^7^ (A) mild (<150 mL/min), (B) moderate (150–450 mL/min) and (C) severe (>450 mL/min).

### Treatment of intraoperative air leaks

**FIGURE 2:**
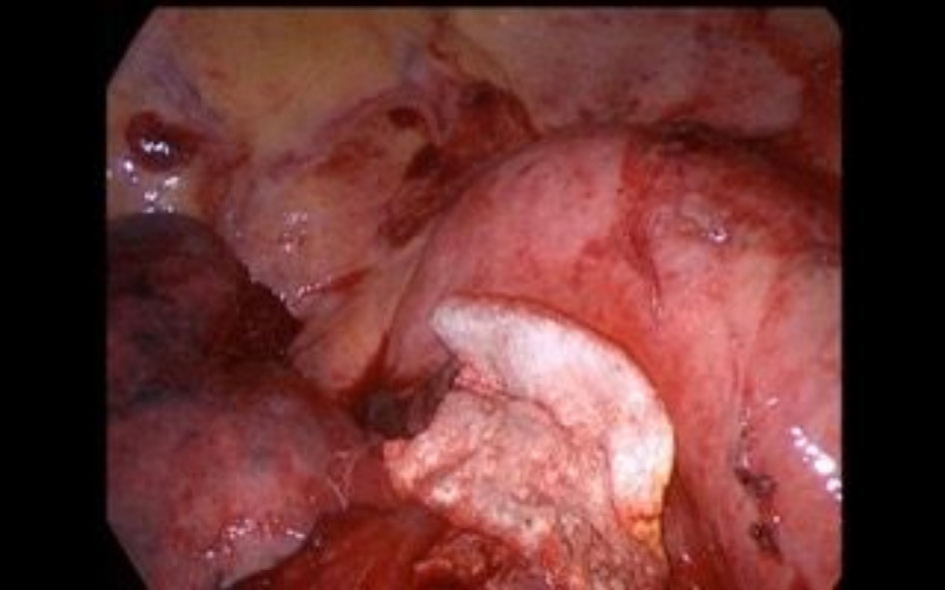
TachoSil is a sponge sealant patch that is coated with active substances consisting of human fibrinogen and human thrombin.

**FIGURE 3:**
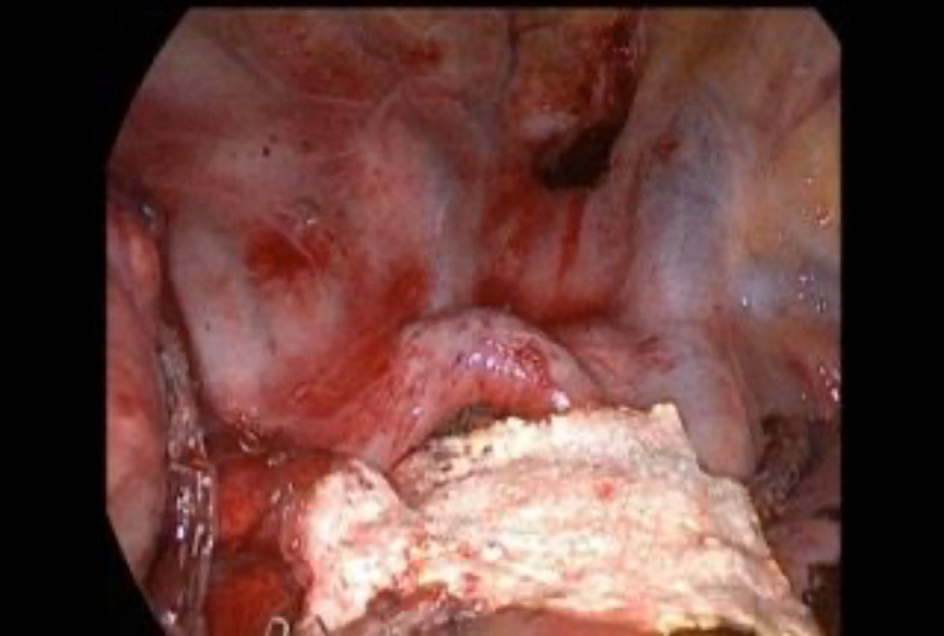
In thoracic cancer TachoSil is used along parenchymal resection and all resection lines and bronchial reconstructions for air tightness.

**FIGURE 4:**
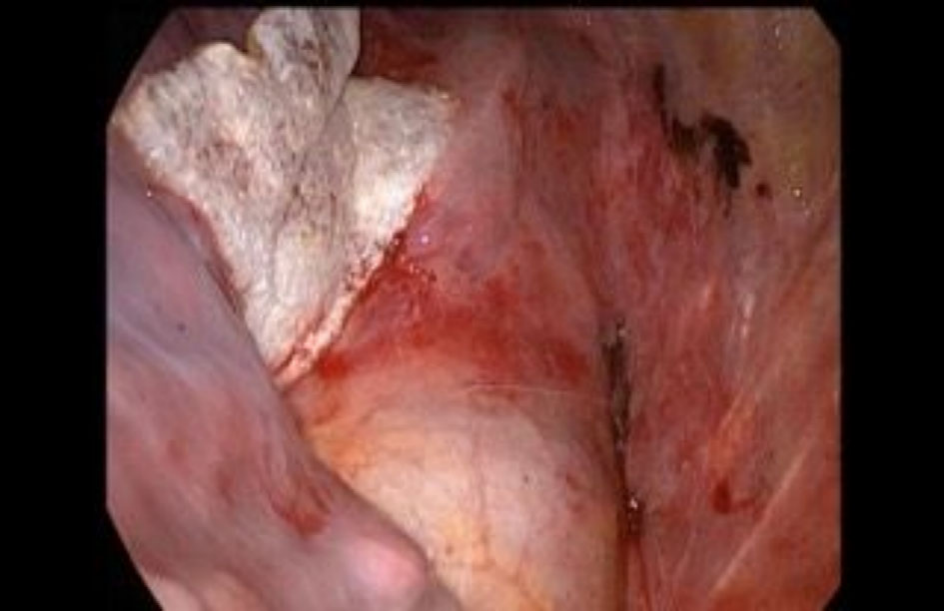
The TachoSil patch is positioned at the site of the lung parenchyma responsible for the air leaks, where it dissolves and disappears completely.

We treated mild IOAALs in a conservative way, whereas for severe IOAAL we looked for the source of leak; if the cause was the bronchial stump, it was sutured and rechecked until air leaks were absent or <150 mL/min. If the source of air leak was from the lung parenchyma, then 4/0 prolene sutures or stapler were used. After the treatment the air leaks were recalculated in order to ensure the reduction was below 150 mL. We decided to use sealant (TachoSil) in patients with IOAAL moderate with cutoff 150–450 mL/min. TachoSil is a sponge sealant patch that is coated with the active substances human fibrinogen and human thrombin (Figure 2). The TachoSil patch is left in the body where it dissolves and disappears completely (Figure 3). We used Tachosil along the parenchymal resection line and along all resection lines including bronchial reconstructions for air sealing tightness (Figure 4). The size of TachoSil to use depends on the length of the lung parenchymal resection line and all bronchial resection and reconstruction lines, so we generally use two sponges (9.5 × 4.8 cm), which can be cut and shaped to depending on the size needed. Once the sponge had been applied to all sites responsible for the identified air leaks, the VMT was repeated and recorded, if the air leaks were still present, TachoSil was applied again (no more than twice). When all large air leaks have been controlled using TachoSil, it is important to observe how much the remaining lung re‐expands to occupy all of the pleural space, aiding complete reapproximation of the visceral pleura to the parietal pleura, further reducing the PAL. We also mobilize the lung, with dissection of the triangular ligament and lysis of the adhesions. TachoSil was not used in severe air leaks because after sealant placement intraoperative persistence of air leaks were seen in these patients, therefore it was necessary to use sutures or staples.

The anesthetist performed the second intraoperative air leak measurement after intraoperative treatment of the air leak. A 28 Fr drainage tube was placed with the tip oriented toward the apex of the pleural cavity, regardless of the lobectomy site, in all patients at the end of the lobectomy and lymphadenectomy. Immediately after surgery, the drainage tubes were placed to water seal.

Postoperatively, the air leak volume (mL/min) was calculated with a digital mass air flow sensing device, and then air leak data were recorded digitally in real‐time. The tube was removed when the lung had sufficiently expanded, with no air leaks, and the amount of liquid drainage was <250 mL/24 h. Therefore, the duration of air leaks after lobectomy surgery was calculated from the day of surgery until chest tube removal.

### Statistical analysis

We compared two groups: Group 1 included 82 patients who underwent VATS lobectomy in which intraoperative air leaks were monitored with water immersion and mechanical ventilation tests; and group 2 which included 73 patients who underwent VATS lobectomy in which intraoperative air leaks were monitored only with a water immersion test. We compared PAL between the two groups, drainage tube duration and hospitalization days. *p*‐values were 0.03, <0.001 and <0.001, respectively. The *p*‐values were considered statistically significant. We used the chi‐square test to perform the statistical analysis. MedCalc statistical software (version 12.3; Belgium) was used for analysis (Table 1).

**TABLE 1:** We compared two groups: a group in which intraoperative air leaks were monitored with the ventilator (group 1) and a group in which intraoperative air leaks were monitored with water immersion test (group 2) and between the two groups PAL, drainage tube duration and postoperative days (POD) were compared.

	Group 1 (*n* = 82)	Group 2 (*n* = 73)	*p*‐value
PAL (%)	2%	10%	0.03
Chest tube removal (mean)	3.45 ± 1.03	4.7 ± 2.03	<0.001
POD (mean)	4.49 ± 1.04	5.9 ± 2.05	<0.001

## RESULTS

This was an observational retrospective single‐center study whose primary aim was to measure intraoperative air leaks with a mechanical ventilation test in order to decide their intraoperative treatment.

We evaluated 82 patients: 50 (61%) males, 32 (39%) females aged 66.4 ± 7.7 years who underwent VATS lobectomy for NSCLC from October 2019 to October 2022 at the Thoracic Surgery Department of the Vanvitelli University of Naples. The inclusion criteria were: age >18 years; no contraindications for surgery. The exclusion criteria were: recent myocardial infarction or unstable angina; severe neurological problems; a prolonged prothrombin time (PT‐INR) >1.5 or a platelet count <30 000; impossibility to tolerate single lung ventilation and pregnancy; benign tumors; pneumonectomy; volume reduction surgery; bulla resection; Pancoast tumors; chest wall or diaphragm resections; sleeve resections and requiring postoperative mechanical ventilation.

There were 53 incomplete fissures and we used linear 75 mm staplers to totally free the fissures. We did not use reinforcement for parenchymal sutures. The postoperative course was uneventful in 79 patients. We observed three complications and one death.

Twenty‐five (30%) patients underwent right upper lobectomy (RUL); three (4%) patients underwent right medium lobectomy (RML), 18 (22%) patients underwent right left lobectomy (RLL); 16 (20%) patients underwent left upper lobectomy (LUL); 18 (22%) patients underwent left lower lobectomy (LLL); one (1%) patient underwent RUL + RML, one (1%) underwent RML + RLL.

Pathology confirmed 49 (60%) patients with adenocarcinoma, 18 (22%) with squamous cell carcinoma; two (3%) with typical carcinoid; three (4%) with atypical carcinoid; two (3%) with large cell neuroendocrine carcinoma and eight (10%) other pathology.

Tumor staging was: 45 patients were T1 (55%); 28 were T2 (34%); seven were T3 (9%); two were T4 (2%); 66 were N0 (80%); 12 were N1 (15%) and four were N2 (5%). In all patients after lobectomy, we evaluated ventilatory leak (VL). VL was diagnosed in 16 (19%) patients. Six patients had a ventilatory leak (VL) < 150 mL/min, they were not receiving any treatment and air leaks resolved spontaneously within 48 h; eight patients had a ventilatory leak (VL) between 150 and 450 mL/min. TachoSil was used in all patients and air leaks resolved within 5 days postoperatively, only one patient presented PAL that resolved, respectively, in 9 days postoperatively, and finally two patients presented VL >450 mL/min. The air leaks were resolved using staplers; however, one patient had persistent air leaks that resolved 13 days postoperatively.

The length of PAL was 10.5 days (range, 8–13 days). All patients were treated conservatively with chest tube drainage. No bronchopleural fistula, pneumonia, atelectasis or respiratory failure was associated with PAL. Two patients (2%) had a minor cardiac complication.

Therefore, PAL incidence was only 2%.

**FIGURE 5:**
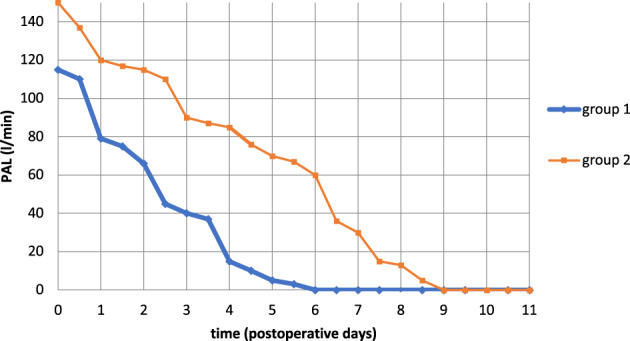
The evaluation of intraoperative air leaks using a mechanical ventilator allows for better management of the same with a reduction in hospitalization days, as summarized in the following graph. Group 1: intraoperative air leaks monitored with the ventilator; group 2: intraoperative air leaks monitored with water immersion test.

The characteristics of the study population are indicated in Table 2. In general, all patients who underwent lobectomy received one 28 F chest tubes. In 82 patients drainage tube removal was performed on 3.45 ± 1.03 days postoperatively and days of hospitalization were 4.49 ± 1.04 days. In our study, we compared PAL, drainage tube duration and hospitalization days between two group: a group in which intraoperative air leaks were monitored with water immersion test and with mechanical ventilation test and a group in which intraoperative air leaks were monitored only with water immersion test (Figure 5). *p*‐values were 0.03, <0.001, <0.001, respectively. The *p*‐values were considered statistically significant.

**TABLE 2:** Patient characteristics.

Variables	Total (*n* = 82)
Gender	
Male	50 (61%)
Female	32 (39%)
Preoperative FEV1 < 60%	2 (3%)
Preoperative FEV1 > 60%	80 (97%)
ppo FEV1 < 60%	12 (15%)
ppo FEV1 > 60%	70 (85%)
Lobe	
RUL	25 (30%)
RML	3 (4%)
RLL	18 (22%)
LUL	16 (20%)
LLL	18 (22%)
RUL + RML	1 (1%)
RML + RLL	1 (1%)
T	
T1	45 (55%)
T2	28 (34%)
T3	7 (9%)
T4	2 (2%)
N	
N0	66 (80%)
N1	12 (15%)
N2	5 (5%)
Histology	
Adenocarcinoma	49 (60%)
Squamous cell carcinoma	18 (22%)
Typical carcinoid	2 (3%)
Atypical carcinoid	3 (4%)
Large cell neuroendocrine carcinoma	2 (3%)
Others	8 (10%)
IOAAL	16 (19%)
<150 mL/min	6 (37.5%)
150–450 mL/min	8 (50%)
>450 mL/min	2 (12.5%)

Abbreviations: IOAAL, intraoperative air leaks; LLL, left lower lobectomy; LUL, left upper lobectomy; RLL, right left lobectomy; RML, right medium lobectomy; RUL, right upper lobectomy.

## DISCUSSION

PAL after pulmonary resection is a severe clinical problem and is an undesirable outcome for thoracic surgeons because it is associated with increased rates, substantial economic costs, prolongation of hospitalization duration, mortality, and other postoperative complications.

After pulmonary lobectomy, PAL is considered to be a leak lasting more than 7 days. This complication occurs in 4%–16% of patients after pulmonary resections.^5^ PALs imply increased hospital costs due to longer hospital stays. This complication is associated with a series of unfavorable consequences such as pneumonia and empyema.^8^ Also, chemotherapy can be delayed if there is a prolonged permanence of the chest drain, moreover infectious complications can interrupt the chemotherapy.^9^ However, some patients are not prepared for this eventuality, in other cases the care facilities are not in a position to accommodate patients with a chest drainage tube, which therefore determines a further delay in discharge.

Several studies in the literature have reported the incidence of PAL after pulmonary lobectomy.^5^

In a review of 369 lobectomies reported by Keagy et al.^10^ there was a 4.3% incidence of PAL; PAL in 4% of patients undergoing lobectomy were reported by D'Andrilli et al.^11^ A correlation between preoperative FEV1 and PAL has been reported in several studies; a higher incidence of PAL in patients with an FEV1/FVC ratio <50% was found by Abolhoda and colleagues.^12^ Several studies have demonstrated that PALs are associated with a reduction in FEV1.^13^, ^14^, ^15^, ^16^ However, a lower incidence of PAL was found in VATS lobectomy compared with thoracotomy lobectomy.^17^, ^18^

A thoracic surgeon is responsible for preventing and controlling air leaks; therefore, his knowledge and experience are essential. Attention to the principles of pneumostasis and precise surgical dissection prevent PALs.

Meticulous and painstaking work during dissection along the anatomical planes while preserving the integrity of the visceral pleura is important in preventing air leakage. Nowadays, staplers are frequently used for the resection of the lung parenchyma, when necessary. The use of a stapler instead of manual dissection for the completion of the fissures is fundamental for the prevention of PALs, as mechanical closure of the lung parenchyma is more precise and pneumostatic.^29^ Fissureless techniques have been associated with a reduced incidence of PAL in both retrospective and randomized studies.^5^, ^19^, ^20^, ^21^, ^22^, ^23^

However, redistribution from the suture lines has also been hypothesized to cause tears in the visceral pleura in areas distant from the suture. Surgical adjuncts have been employed for PAL prevention, such as suture line reinforcement, pleural tenting, mechanical pleurodesis, pneumoperitoneum,^23^, ^24^, ^25^, ^26^, ^27^, ^28^ and biological and synthetic sealants are increasingly common methods for PAL prevention.^29^, ^30^

Our method for accurately quantifying air leaks intraoperatively uses the flowmeter attached to modern anesthesia machines without interrupting mechanical ventilation.^31^

Intraoperative VL monitoring is recommended to provide real‐time intraoperative guidance to assess whether surgical additions are needed or if simple repair would be sufficient. Therefore, unlike the pre‐ and intraoperative risk factors mentioned which cannot be changed during surgery, the VL can be reduced with further intraoperative interventions.^32^

In our experience, the risk factors responsible for air leaks due to surgery are considered more closely related to immediate postoperative and prolonged air leaks, therefore the calculation of IOAALS could prevent PALs.

The traditional technique for assessing intraoperative air leaks is to pour saline into the thoracic cavity filling it, and then observing the degree of air bubbles, which form during manual positive pressure ventilation. This method is extremely subjective and depends on the experience of the individual surgeon; however, our method allows the detection of intraoperative VL using the flowmeter connected to modern anesthesia machines, quantifying air leaks without suspending mechanical ventilation.^7^ Considering the postoperative PAL based on the results of our study, we can provide some recommendations: use extra caution in patients with risk factors such as male sex (*p* = 0.043), history of smoking ≥40 pack‐years (*p* = 0.021), preoperative serum albumin level ≤4.0 mg/dL (*p* = 0.003)^33^ open thoracotomies (*p* = 0.081), anatomical resections (*p* < 0.001), the presence of pleural adhesions^34^ at the time of the operation (*p* = 0.032), reduced parameters of FEV1 (*p* < 0.001) and DLCO (*p* < 0.001), operations in the right hemithorax (*p* = 0.0092), the surgical site (OR = 2.71) are associated with an increased risk of developing a sustained air leak,^13^, ^14^ but we particularly recommend real‐time intraoperative VL monitoring as an intraoperative guide.

In our study, we calculated the intraoperative air leak after lobectomy^7^ and based on the values reported by mechanical ventilation test and the established cutoff we tried to apply in real‐time intraoperatively the most suitable treatment to prevent PALs.^11^

The study is not without limitations, which include its retrospective character and the fact that it only collects the experience of a single center.

In conclusion, nowadays thoracic surgeons are aware of iatrogenic complication and are highly motivated to find more effective strategies to prevent PALs. Treatment of intraoperative air leaks involved a significant reduction in intrapleural drainage time and days of hospitalization. The VL measurement during surgery may be useful for deciding whether or not to use surgical adjuncts for the treatment of air leaks during pulmonary resection and evaluating the effects of the treatment immediately. There are several advantages to rapid removal of the drainage tube: faster mobilization, a reduction in pain, a shorter period of hospitalization and consequently savings on hospital costs.

Thus, we showed that VL at the end of lobectomy is a quantitative and objective measure of air leaks after lung resection; it can be considered an independent predictor of PAL providing real‐time intraoperative guidance to identify those patients who may benefit from intraoperative preventive interventions to reduce PALs. However, studies of larger patient populations are needed to corroborate our hypotheses.

## AUTHOR CONTRIBUTIONS

Conceptualization, G.M. and G.N.; Methodology, M.B., M.M., and E.M.; Software, G.O. and G.V.; Validation, G.L., R.F., and M.C.; Formal analysis, B.L. and M.G.; Investigation, V.D.F. and R.M.; Resources, F.C. and D.G.P.; Data Ccration, F.P.D. and A.N.; Writing–original draft preparation, G.M.; Writing–Review and editing, M.B.; Visualization, M.F.; Supervision, A.F.; Project administration, G.V.; Funding acquisition, F.C. and M.F.

## CONFLICT OF INTEREST STATEMENT

The authors declare no conflict of interests.

## Data Availability

Data are contained within the article.
